# Supplementary material for: Equilibrium and non-equilibrium furanose selection in the ribose isomerisation network
Source: Nat Commun. 2021 May 12;12:2749. doi: 10.1038/s41467-021-22818-5 (PMC8115175; doi:10.1038/s41467-021-22818-5)
Supplement: Supplementary file 1 — Supplementary Information [file 41467_2021_22818_MOESM1_ESM.pdf]

Equilibrium and non-equilibrium furanose selection in the ribose isomerisation network. Supplementary information

CONTENTS

Supplementary Note 1. NMR: Choice of the relaxation time T_1 in the NMR experiments 3

Supplementary Note 2. Extracting the equilibrium molar fractions from the NMR spectra 3

Supplementary Note 3. Model Hadean water 4

Supplementary Note 4. Diagrammatic calculation of the steady-state molar fractions 8
A. The limit $k_D \to 0$, thermal equilibrium 11
B. Finite values of the transport rate k_D, non-equilibrium steady states 11

Supplementary Note 5. Analytical expressions for the two crossover rates 15
C. The first crossover 15
D. The second crossover 17

Supplementary Note 6. Cycle fluxes 18

Supplementary Note 7. Rate of entropy production and energy dissipation 19
E. The limit of vanishing transport rate 20

Supplementary References 25

SUPPLEMENTARY NOTE 1. NMR: CHOICE OF THE RELAXATION TIME T_1 IN THE NMR EXPERIMENTS

The first step in order to quantify the relative population of each anomer through ^{13}C NMR experiments is to evaluate the relaxation time T_1 for all species. Since T_1 is strongly dependent on temperature, T_1 measurements were carried out at each temperature before the spectral data were recorded. The results of these experiments are reported in Table 1. It can be appreciated that T_1 increases significantly with temperature from about 2 to 5 seconds, for temperatures between 10 °C and 80 °C, the temperature range of this study. Overall, however, it is fair to say that the values of T_1 are similar for all anomers at a given temperature. Only αpyranose shows a significantly higher value at the higher temperature of 80 °C. Building on these results, we investigated the anomerisation of ribose by ^{13}C NMR at different temperatures, by adapting the D_1 ($5T_1$) for each experiment.

Supplementary Table 1. Values of T_1 (seconds) for C1 of ribose at thermal equilibrium at different temperatures.

Temperature (°C)	αP	αF	βP	βF
20	1.99	1.59	1.79	1.87
30	2.37	2.85	2.42	2.15
40	2.74	3.62	2.77	2.91
50	3.77	3.56	3.51	3.74
60	5.06	5.49	5.19	5.10
70	5.79	6.44	5.11	5.18
80	7.94	5.16	6.11	5.79

SUPPLEMENTARY NOTE 2. EXTRACTING THE EQUILIBRIUM MOLAR FRACTIONS FROM THE NMR SPECTRA

At 25 °C (T_1 = 1.8 s), the signals of C1-αP, C1-βP, C1-αF and C1-βF are found, respectively, at 93.56 ppm, 93.85 ppm 96.33 ppm and 100.99 ppm. A large number of ^{13}C NMR spectra, between 50 and 80, was recorded for each temperature and the average relative molar fractions computed by fitting the corresponding peak areas with Lorentzian line shapes.

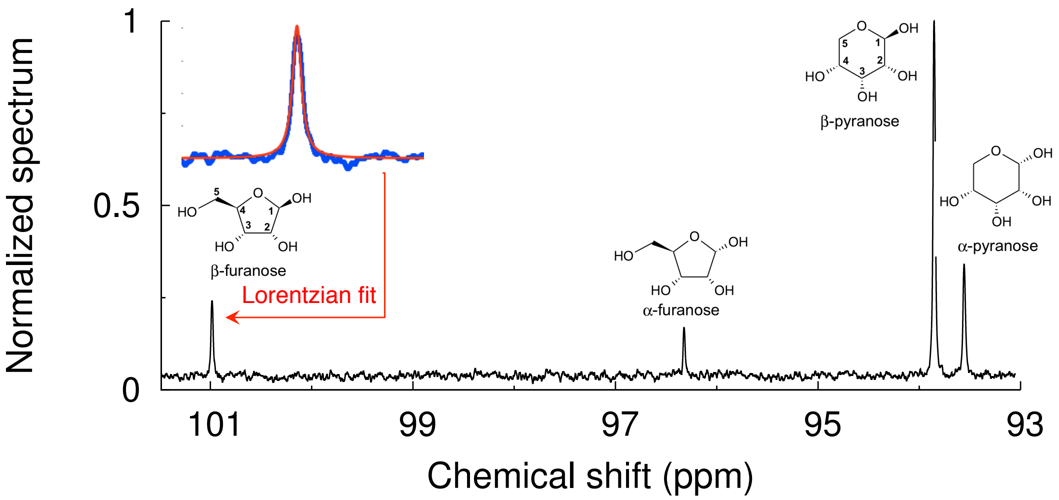

Supplementary Figure 1. ^{13}C NMR spectrum of D-ribose in solution at $T = 25$ °C and ambient pressure. The relative fractions of the four species x_i were estimated by fitting the areas A_i below the respective NMR peaks, i.e. $x_i = A_i / \sum_m A_m$. The inset shows a typical Lorentzian fit of one of the anomers' peak.

This procedure is illustrated for one representative spectrum in Fig. 1.

SUPPLEMENTARY NOTE 3. MODEL HADEAN WATER

The composition of the artificial sea water [1] used in our NMR experiments is reported in Table 2.

- NaCl in the modern ocean is 35g/L, and has maximum values at the vents of hydrothermal systems. Fluid inclusion study demonstrates that the concentration of NaCl exceeds current levels in both Archaean seawater and hydrothermal fluid. Zharkov [2] supported an Archaean sea of 1.2 times the current salinity, inferring lacking complexity of biological evolution. This was refuted by Knauth [3], who advocated an ocean at least 1.6 times as saline as the modern. de Ronde et al. [4] conducted detailed fluid inclusion studies in Barberton Greenstone Belt ironstones to assign ionic concentrations to the water in which those formed. We have selected the hydrothermal end-member of their dataset as being the example which most closely represents the environment we aim to simulate. Therefore, the Archaean ocean at the sediment-hydrothermal water interface is approximately twice the salinity of the modern ocean, 70 g/L. Rollinson [5]

Supplementary Table 2. Salt composition of the simulated Hadean sea water used in our ^{13}C NMR experiments, pH 6.3.

Compound	g/L
NaCl	70.00
$NaSiO_2$	0.30
$FeCl_2$	0.30
KCl	1.40
$NaHCO_3$	0.10
KBr	0.30
H_3BO_3	0.20
NaF	0.50
$MgCl_2$. $6H_2O$	19.30

suggests that the Archaean ocean was saturated in both the ions of Na and Cl.

- $NaSiO_2$ is added to provide the Si ion. The continued addition of Na is inconsequential, as explained above it was oversaturated. Knauth [6] suggests that in an ocean of 50 °C, Si would have a concentration of 300ppm, however, Rimstidt [7] had previously given a conservative estimate of only 20ppm. Early Archaean stratigraphy is profoundly and ubiquitously silicified throughout, particularly in the hydrothermally influenced shallow water environments which we aim to simulate in this experiment. Therefore, we chose the value of 300ppm (0.3 g/L), with the observation that this is perhaps even a conservative estimate of the waters. Knauth's estimation was for the whole ocean; unfortunately, the modern hydrothermal analogues are not strictly equivalent to ancient examples.

- $FeCl_2$ was chosen to replace the $FeSO_4$ of the modern ocean, and is specifically to add Fe; as mentioned above, the saturation of the ocean in Cl [5] means that its overaddition should not affect our estimation. SO_4 was low to negligible in the Hadean-Archaean ocean [8, 9], but there was a substantial Fe dissolved reservoir [10, 11]; Fe in Archaean carbonate indicates that concentrations in the equilibrating ocean were twice their current values. We consider the example of Lac Pavin, France, a supposed

Archaean analogue water body featuring an iron system [12]. Throughout the lake profile, Fe concentrations vary from 2 μM in the top waters to 2,400 μM in the iron-rich waters at the base; we select the latter value as representative of the hydrothermal scenario that we propose. This value is similar to those of Archaean and modern hydrothermal zone Fe concentration described by Douville et al. [13–15]. It exceeds the 40-120 μM suggested by Canfield [10, 16, 17], though their estimations represent oceanic concentrations, which we consider to be the seawater end-member.

- KCl is added to provide the K ion, which would have been sourced from effusions of hydrothermal fluids and dissolution of feldspar. Rollinson [5] suggests that K concentration was 19 mM in the Archaean ocean. We consider that this estimate may be conservative, however, there would have been a limited flux from erosion of the seafloor which, being komatiite-basalt in composition, has low, but not negligible, K content.

- $NaHCO_3$ provides the bicarbonate, and Na, which was saturated. Using the relationships of dissolved carbonates in the ocean, we conclude that at pH 6, 70 % (0.7 mole fraction) of total carbonate would have been bicarbonate. Thus, of the three phases in the carbonate diagram, bicarbonate would be the dominant phase. The presence of carbonates in the Archaean is understood only poorly due to a paucity in their preservation. We have used the pre-industrial value of bicarbonate, 1, 757 μM/L, to ensure that the ion is present, though this is the least well-constrained component.

- KBr is added for the Br ion which, from fluid inclusion studies of 3.23 Ga ironstone pods in Barberton, is estimated at 2.59 mM/L for the hydrothermal end-member. A corollary of this addition is the addition of K, which has already been accounted for in KBr.

- H_3BO_3 is chosen for the BO_3 ion. Modern values are 4-5 ppm (0.005 g/L), which we take as the absolute minimum for Archaean values. Since borate is associated with hydrothermal activity, we have taken the values of current hydrothermal fluids at 203 ^{o}C as indicative of the levels of borate present in our simulated hydrothermal scenario. Therefore, 0.2 g/kg of the compound is needed [18].

- NaF is added for the F ion, which is major in hydrothermal effusions. No data for F

was extracted from the ironstone fluid inclusions of de Ronde et al. [4], no from any other example in our extensive literature survey. We thus turned to modern submarine hydrothermal systems as the closest available analogue. Values include 44 μM/L, 36.2-65.4 μM (of which the 40.2-42.9 μM bracket (cool flange) may be most representative: island arc hydrothermal field, and 500 ppm in an ore-forming fluid (since this fluid is an ore-forming example, the value is likely a slight overestimation of our more passive hydrothermal scenario).

- $MgCl_2.6H_2O$ is added for Mg, and since the ultramafic oceanic crust was rich in olivine-containing rocks (komatiite and tholeiite), we have taken the upper limit estimated by Rollinson [5] of 95 mM/L.

- $CaCl_2.2H_2O$ is added for Ca, and since pyroxene was a major mineral in the reactive (dissolution of volcanic glass and feldspar) ultramafic oceanic crust, we have taken the upper limit estimate by Rollinson [5] of 50 mM/L.

Theory

In the following, the energy of furanose (F) is referred to as E_1, that of pyranose (P) as E_2, while that of the transition state, linear sugar (L) is referred to as E_0.

SUPPLEMENTARY NOTE 4. DIAGRAMMATIC CALCULATION OF THE STEADY-STATE MOLAR FRACTIONS

With reference to the node numbering shown in Fig. 2, the rate equations associated with the reduced reaction network (one high-energy (F) state E_1, one low-energy (P) state E_2 and a transition state, E_0) read

$$\begin{aligned}
\dot{x}_0 &= k_{10}x_1 + k_{50}x_5 + k_D x_3 - (k_{01} + k_{05} + k_D)\, x_0 \\
\dot{x}_1 &= k_{01}x_0 + k_D x_2 - (k_{10} + k_D)\, x_1 \\
\dot{x}_2 &= k_{32}x_3 + k_D x_1 - (k_{23} + k_D)\, x_2 \\
\dot{x}_3 &= k_{23}x_2 + k_{43}x_4 + k_D x_0 - (k_{32} + k_{34} + k_D)\, x_3 \\
\dot{x}_4 &= k_{34}x_3 + k_D x_5 - (k_{43} + k_D)\, x_4 \\
\dot{x}_5 &= k_{05}x_0 + k_D x_4 - (k_{50} + k_D)\, x_5
\end{aligned} \tag{1}$$

with the normalisation $\sum_{m=0}^{5} x_m = 1$. The explicit expressions for the rates read (see also main text):

$$\begin{aligned}
k_{01} &= \mu\, e^{-\beta_2 \Delta E_1} & k_{32} &= \mu\, e^{-\beta_1 \Delta E_1} \\
k_{05} &= \mu\, e^{-\beta_2 \Delta E_2} & k_{34} &= \mu\, e^{-\beta_1 \Delta E_2} \\
k_{10} &= \frac{\mu}{\eta_1}\, e^{-\beta_2 (E_0 - E_1 + \Delta E_1)} & k_{23} &= \frac{\mu}{\eta_1}\, e^{-\beta_1 (E_0 - E_1 + \Delta E_1)} \\
k_{50} &= \frac{\mu}{\eta_2}\, e^{-\beta_2 (E_0 - E_2 + \Delta E_2)} & k_{43} &= \frac{\mu}{\eta_2}\, e^{-\beta_1 (E_0 - E_2 + \Delta E_2)}
\end{aligned} \tag{2}$$

where $\beta_i^{-1} = k_B T_i$, $i = 1, 2$. Following Ref. 19, the stationary state of the rate equations (1) can be computed by referring to the complete set of *partial diagrams* of the network (see Fig. 2). The steady-state value of state m is then obtained by introducing a directionality to each line in each partial diagram, so that all connected path *flow* toward the vertex m. Each directional line corresponds to a rate constant, hence each *directional* partial diagram corresponds to the product of five rate constants. The prescription is then that each x_m is proportional to the sum of all the associated directional diagrams. It will be remarked that the partial diagrams contain either 1,2 or 3 *mobility* link, and therefore they can be

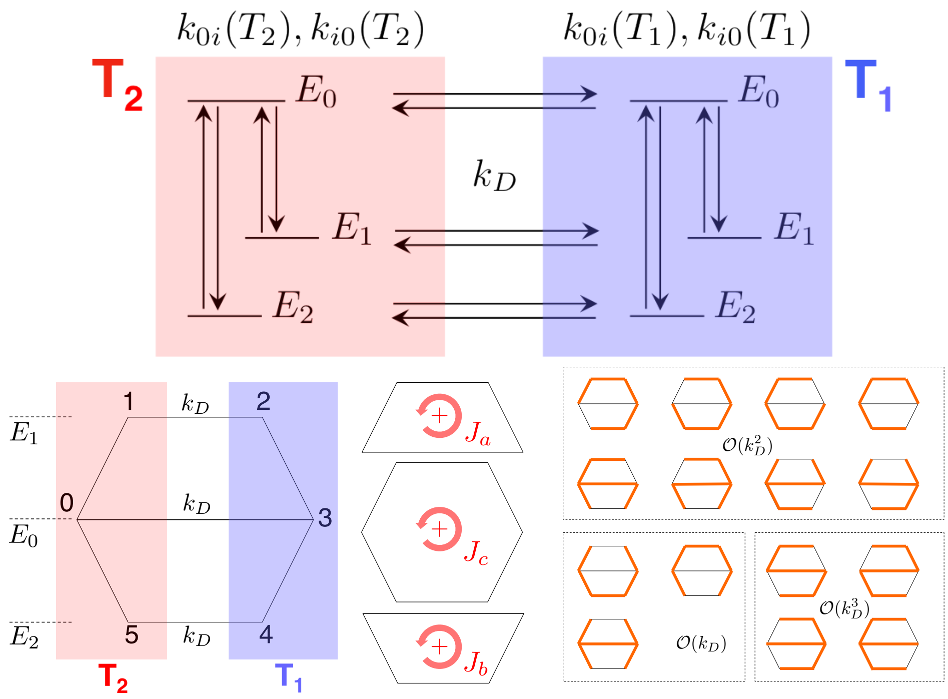

Supplementary Figure 2. (Top) reduced model of ribose isomerisation reactions in a steady temperature gradient ($T_2 > T_1$). The highest-energy linear species has energy E_0. The two furanose species have been coalesced into the high-energy species 1 (energy E_1), while the two pyranose species represent the ground state in this model. Each species can diffuse or be advected across the temperature gradient with a global rate k_D. Bottom left: Graph corresponding to the above chemical network with identification of the nodes and illustration of the three cycle fluxes present in this model. The convention for positive cycles is shown explicitly. Bottom right: the complete set of partial diagrams, each of which contains the maximum number of lines (five here) that can be included without forming a cycle. The partial diagrams are grouped according to the number of *mobility* links that they contain (i.e. branches giving a contribution proportional to k_D).

classified as corresponding to terms of order k_D^n ($n = 1, 2, 3$ in the expressions for the stationary probabilities (molar fractions). As an example, considering state 1, the steady-state probability reads

$$x_1 = \frac{k_D^3(k_{43}k_{01} + k_{50}k_{32}) + k_D^2(k_{43}k_{32}k_{01} + [\dots]_7) + k_D(k_{50}k_{01}k_{23}k_{34} + [\dots]_2)}{k_D^3(k_{43}k_{01} + k_{50}k_{32}) + k_D^2(k_{43}k_{32}k_{01} + [\dots]_7) + k_D(k_{50}k_{01}k_{23}k_{34} + [\dots]_2) + [\dots]_{65}} \quad (3)$$

where the notation $[\dots]_n$ stands for n *other* terms. Overall, each expression such as eq. (3) contains 13 terms in the numerator (i.e. as many as there are partial diagrams) and 78 terms in the denominator (i.e. 13 terms multiplied 6 nodes). As stated in the main text,

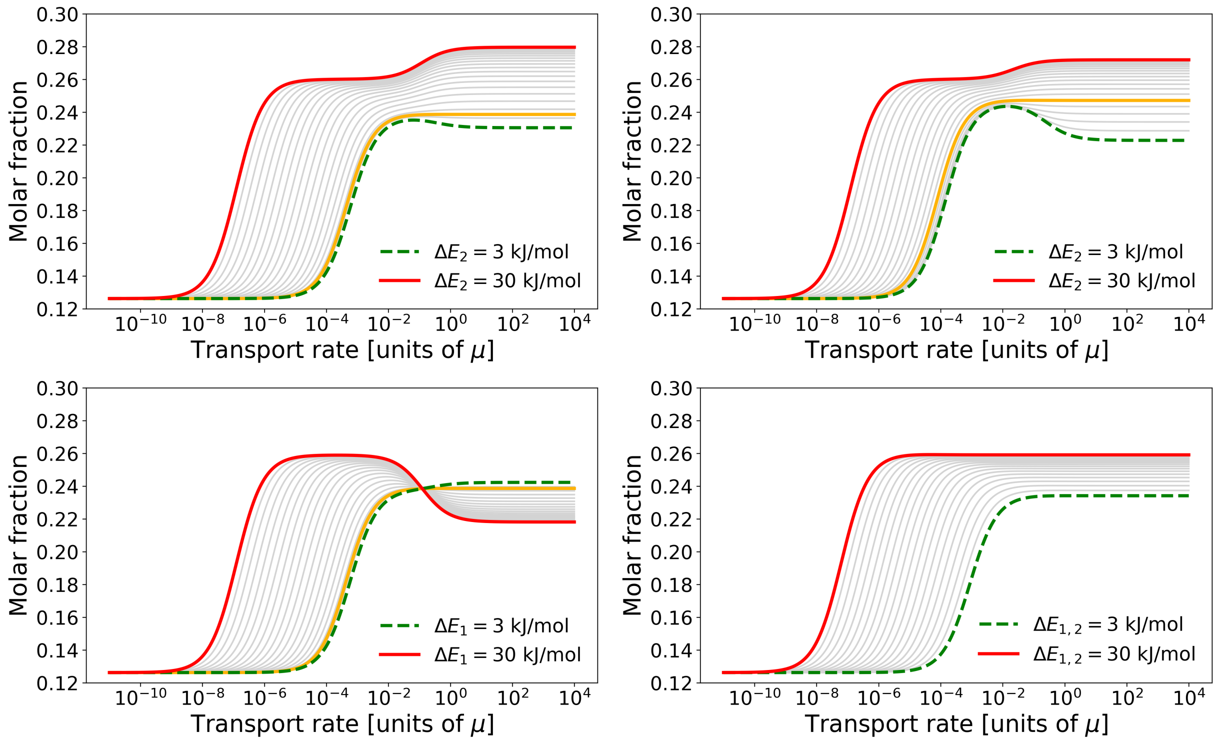

Supplementary Figure 3. **Illustration of the ultra-stabilisation/ultra-destabilisation effects**. Molar fraction of the high-energy furanose species in a steady thermal gradient of the same order as that present between the top and the base of a thermal vent ($T_2 = 210$ °C, $T_1 = 60$ °C) as a function the transport rate k_D (in units of $\mu = k_{01}^{\infty} = k_{02}^{\infty}$) for different choices of the energy barriers $\Delta E_1, \Delta E_2$. Clockwise from top to bottom: $\Delta E_1 = 5$ kJ/mol and varying $\Delta E_2 \in [3, 30]$ kJ/mol, $\Delta E_1 = 10$ kJ/mol and varying $\Delta E_2 \in [3, 30]$ kJ/mol, $\Delta E_2 = 5$ kJ/mol and varying $\Delta E_1 \in [3, 30]$ kJ/mol (the amber lines correspond to $\Delta E_2 = \Delta E_1$), varying $\Delta E_1 = \Delta E_2 \in [3, 30]$ kJ/mol. Parameters are $\eta_1 = 29.34$, $\eta_2 = 1.945$, $E_0 = 19$ kJ/mol, $E_1 = 13.6$ kJ/mol, $E_2 = 3.1$ kJ/mol, corresponding to the average values for the two furanose and pyranose enantiomers measured from equilibrium NMR experiments (see Table I in the main text).

such expressions can be cast in the general form of ratios of second-order polynomials, i.e.

$$x_i = \frac{\alpha_{2i} k_D^2 + \alpha_{1i} k_D + \alpha_{0i}}{A_2 k_D^2 + A_1 k_D + A_0}, \qquad i = 0, 1, \ldots, 5 \tag{4}$$

with

$$A_n = \sum_{j=0}^{5} \alpha_{nj}, \qquad n = 0, 1, 3 \tag{5}$$

A. The limit $k_D \to 0$, thermal equilibrium

In our model, the thermal equilibrium scenario corresponds to two isolated boxes, each reaching thermal equilibrium at a separate temperature. Mathematically, this corresponds to the limit $k_D \to 0$, $\mathrm{Da} \propto k_D^{-1} \to \infty$, the steady-state populations reducing to their respective equilibrium expressions (see Eqs. (2) in the main text),

$$x_i^{\mathrm{eq}} \stackrel{\mathrm{def}}{=} \lim_{k_D \to 0} x_i = \frac{\alpha_{0i}}{A_0} \tag{6}$$

More precisely, we have

$$\begin{aligned} x_1^{\mathrm{eq}} &= \tfrac{1}{2} P_{E_1}^{\mathrm{eq}}(T_2) \quad x_2^{\mathrm{eq}} = \tfrac{1}{2} P_{E_1}^{\mathrm{eq}}(T_1) \\ x_5^{\mathrm{eq}} &= \tfrac{1}{2} P_{E_2}^{\mathrm{eq}}(T_2) \quad x_4^{\mathrm{eq}} = \tfrac{1}{2} P_{E_2}^{\mathrm{eq}}(T_1) \\ x_0^{\mathrm{eq}} &= \tfrac{1}{2} P_{E_0}^{\mathrm{eq}}(T_2) \quad x_3^{\mathrm{eq}} = \tfrac{1}{2} P_{E_0}^{\mathrm{eq}}(T_1) \end{aligned} \tag{7}$$

with

$$P_{E_i}^{\mathrm{eq}}(T) = \frac{\eta_i e^{-E_i/k_B T}}{Z(T)}, \tag{8}$$

where $Z(T) = e^{-E_0/k_B T} + \eta_1 e^{-E_1/k_B T} + \eta_2 e^{-E_2/k_B T}$ denotes the partition function at temperature T. The factor 1/2 in Eqs. (7) reflects the fact that each box contains half of the total mass.

B. Finite values of the transport rate k_D, non-equilibrium steady states

As transport of reactants across the temperature gradient is put back into the picture, non-equilibrium effects start shifting the steady state away from thermal equilibrium, as sustained currents set in, coupling mass transport to chemical transformations. Fig. 4 shows clearly that, when production of furanose proceeds faster than pyranose (i.e. $\Delta E_2 > \Delta E_1$), this causes the population of furanose to grow beyond its highest equilibrium value at values of Da of order one. We refer to this non-equilibrium effect as *ultra-stabilisation* of furanose.

More precisely, with the choice of parameters derived from our NMR experiments, the steady-state solutions (4) display three regimes as a function of the transport rate k_D, identified by two distinct crossovers occurring at characteristic rates $k_D^{*[1]}$ and $k_D^{*2} \geq k_D^{*[1]}$, as illustrated in Fig. 4. These can be computed straightforwardly from Eq. (4) and show a marked dependence on the choice of the energy barriers ΔE_1 and ΔE_2, as illustrated by Fig. 5. However, with the choice of parameters extracted from our equilibrium experiments,

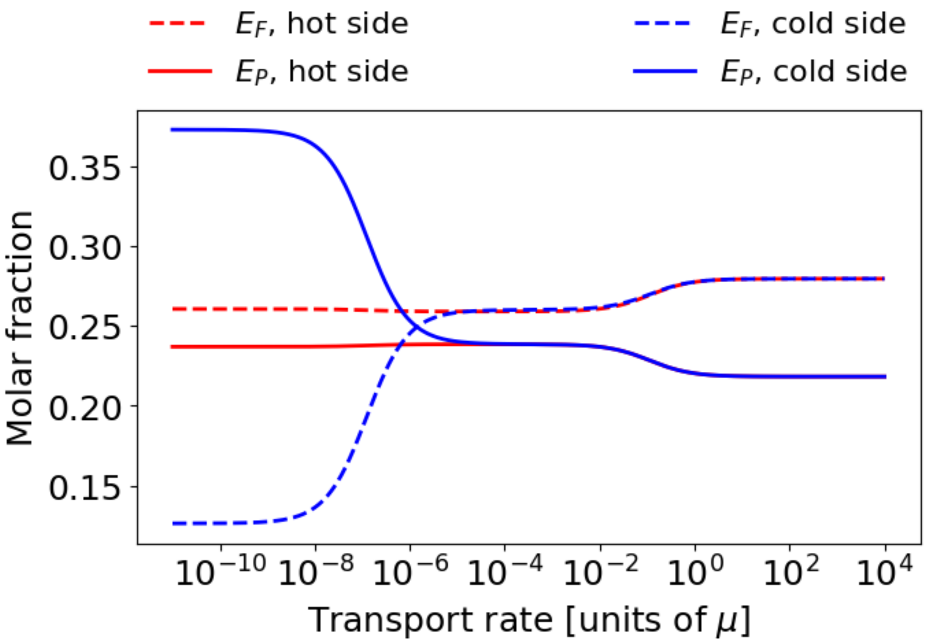

Supplementary Figure 4. **The high-energy furanose species can be ultra-stabilised by sustained mass currents at large enough transport rates**. Molar fraction of furanose (dashed lines) and pyranose (solid lines) species in a steady thermal gradient with $T_2 = 210\,^\circ$C, $T_1 = 60\,^\circ$C vs transport rate k_D in units of μ (Da^{-1}) for $\Delta E_1 = 5$ kJ/mol, $\Delta E_2 = 30$ kJ/mol. Two consecutive stabilisation crossovers are observed as the transport rate increases, the final *ultra-stabilisation* transition occurring when Da $\simeq 1$ ($k_D \simeq \mu$). Equilibrium parameters are $\eta_1 = 29.34$, $\eta_2 = 1.945$, $E_0 = 19$ kJ/mol, $E_1 = 13.6$ kJ/mol, $E_2 = 3.1$ kJ/mol, set as the average values for the two furanose and pyranose enantiomers measured from equilibrium NMR experiments (see Table I in the main text).

it turns out that the two crossovers are always distinct, $k_D^{*[2]}$ being at least three orders of magnitude larger than $k_D^{*[1]}$ irrespective of the magnitude of the energy barriers.

Increasing k_D past $k_D^{*[1]}$, the steady-state populations of same-species states become independent of the temperature gradient. In this regime, the molar fractions at the cold side become identical to the values at the hot side and the two boxes can be regarded as a well-stirred open chemical reactor. This is due to a small, but non-zero steady mass current that circulates in the direction $0 \to 1 \to 2 \to 3 \to 4 \to 5 \to 0$ (see Fig. 2) and hence only involves transport of closed-ring species across the temperature gradient in the hot-to-cold direction. Fig. 5 (left) shows that this first non-equilibrium effect occurs in a regime

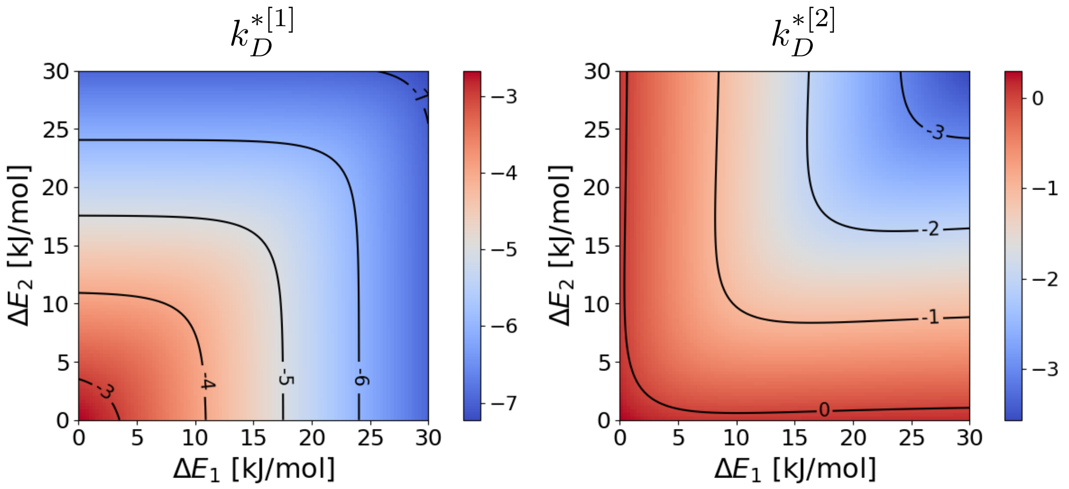

Supplementary Figure 5. **The mobility rates that mark stabilisation crossovers of furanose are well separated and depend markedly on the energy barriers**. Density plot of $k_D^{*[2]}$ and $k_D^{*[1]}$ (in units of μ) in the plane $\Delta E_1, \Delta E_2$ in logarithmic (base 10) scale. Parameters as in Fig. 4 (see Table I in the main text)

where mass transport is still much slower than chemical reactions, i.e. for values of Da $= \mu/k_D > 10^3$. Our calculations show that sustained currents that stabilise the high-energy species kick in at exponentially slower transport rates as the energy barriers increase. More precisely, it can be shown that (see the following)

$$k_D^{*[1]} \simeq \frac{\mu}{P_{E_1}^{\text{eq}}(T_1)\left[f_1\left(e^{\Delta E_1/k_BT_1}+e^{\Delta E_2/k_BT_1}\right)+f_2\left(e^{\Delta E_1/k_BT_2}+e^{\Delta E_2/k_BT_2}\right)\right]} \tag{9}$$

where $f_i = P_{E_2}^{\text{eq}}(T_i)/P_{E_0}^{\text{eq}}(T_i)$. In other words, when the energy barriers are small, hence chemical steps faster, molecular species need to diffuse or be advected faster to settle into a different steady state than at thermal equilibrium.

When the mobility rate increases past $k_D^{*[1]}$, a second crossover to a different steady state is seen to occur at $k_D \simeq k_D^{*[2]}$. We find that, when the two energy barriers are well separated, i.e. $\Delta E_1 \ll \Delta E_2$ or $\Delta E_2 \ll \Delta E_1$, this second crossover corresponds to a Damköhler number $\text{Da}^* = \mu/k_D^{*[2]} \simeq \mathcal{O}(1)$, provided the smaller barrier does not exceed the average thermal energy provided at the hot end, k_BT_2. This is the fast-transport regime, where transport and chemical reaction time scales match (see Fig. 5, right). The relation between the critical value of the Damköhler number and the main determinants of relaxation kinetics, i.e. the

energy barriers, can be encapsulated in a remarkably transparent formula, notably

$$\mathrm{Da}^* = \frac{1}{e^{-\Delta E_1/k_B T_M} + e^{-\Delta E_2/k_B T_M}} \tag{10}$$

where we have indicated with $T_M = (T_1 + T_2)/2$ the average temperature of the system. From Eq. (31) it can be readily seen that the requirement for kinetic selection, i.e. scenarios where one of the barriers is much higher than the average temperature and the other much lower, indeed correspond to the timescale-matching condition $\mathrm{Da}^* = \mathcal{O}(1)$.

The second crossover is seen to occur at the same value of Da irrespective of whether production of furanoses is faster than of pyranoses or viceversa. However, the physical consequences of this second transition turn out to be strikingly divergent, depending on which of the two barriers is the largest and on their relative magnitude. If furanose production is the fastest relaxation channel, i.e. $\Delta E_1 \ll \Delta E_2$, the second crossover leads to ultra-stabilisation of the most unstable closed form of the sugar [1], as sustained currents keep the population of furanose beyond the highest value accessible at equilibrium, $P_{E_1}^{\mathrm{eq}}(T_2)$. It can be shown that the relative furanose-to-pyranose population takes a remarkably simple expression in the ultra-stabilisation regime

$$\frac{P_{E_1}^{\infty}}{P_{E_2}^{\infty}} \simeq \frac{P_{E_1}^{\mathrm{eq}}(T_2)}{P_{E_2}^{\mathrm{eq}}(T_2)} e^{(E_0 - E_1)\Delta T/k_B T_1 T_2} \tag{11}$$

where $\Delta T = T_2 - T_1$ is the temperature gradient. Eq. (11) conveys an important piece of physical information. The relative dissipation-sustained enrichment of the high-energy furanose species, E_1, is magnified (exponentially) the larger its energy separation from the high-energy intermediate (linear) state, E_0. Of course, the larger the temperature gradient, the greater this effect.

In general, it not difficult to check that the condition for the two-crossover trend observed in Fig. 4 to exist is

$$A_1 \gg \sqrt{A_0 A_2} \tag{12}$$

In this case, the functions x_i will display a three-plateau trend as follows

$$x_i(k_D) \simeq \begin{cases} \dfrac{\alpha_{0i}}{A_0} & \text{for} \quad k_D \ll k_D^{*[1]} \\ \dfrac{\alpha_{1i}}{A_1} \overset{\mathrm{def}}{=} x_i^{\mathrm{int}} & \text{for} \quad k_D^{*[1]} \ll k_D \ll k_D^{*[2]} \\ \dfrac{\alpha_{2i}}{A_2} & \text{for} \quad k_D \gg k_D^{*[2]} \end{cases} \tag{13}$$

[1] Conversely, if $\Delta E_1 \gg \Delta E_2$, pyranose production production proceeds much faster, and it is that species which is ultra-stablised.

where the two crossover mobility rates are given by

$$k_D^{*[1]} = \frac{A_0}{A_1} \qquad k_D^{*[2]} = \frac{A_1}{A_2} \tag{14}$$

With the choice of parameters extracted from our NMR experiments, the second crossover corresponds to a transport rate that is at least 3 orders of magnitude larger than that marking the first crossover (see Fig. 6).

Furthermore, with the following definitions, which mark the relevant populations that characterise the three different regimes (int for intermediate regime),

$$x_i^{\text{eq}} \overset{\text{def}}{=} \lim_{k_D \to 0} x_i(k_D) = \frac{\alpha_{0i}}{A_0} \tag{15}$$

$$x_i^{\text{int}} \overset{\text{def}}{=} \frac{\alpha_{1i}}{A_1} \tag{16}$$

$$x_i^{\infty} \overset{\text{def}}{=} \lim_{k_D \to \infty} x_i(k_D) = \frac{\alpha_{2i}}{A_2} \tag{17}$$

the steady-state populations, Eq. (4), can be cast in the more transparent form (see again Eq. (14))

$$x_i(k_D) = \frac{x_i^{\infty} k_D^2 + x_i^{\text{int}} k_D^{*[2]} k_D + x_i^{\text{eq}} k_D^{*[1]} k_D^{*[2]}}{k_D^2 + k_D^{*[2]} k_D + k_D^{*[1]} k_D^{*[2]}} \tag{18}$$

SUPPLEMENTARY NOTE 5. ANALYTICAL EXPRESSIONS FOR THE TWO CROSSOVER RATES

An analytical approximation of the crossover rates $k_D^{*[1]}$ and $k_D^{*[2]}$ can be obtained by noticing that the same crossovers are observed for the molar fractions of the same species in either box as functions of the transport rate k_D. Hence, the abscissae of the (logarithmic) inflection points can be estimated by considering ratios between same-species molar fractions.

C. The first crossover

In order to compute $k_D^{*[1]}$, it is expedient to focus on the high-energy species (nodes 1 and 2 in the graph, corresponding to T_2 and T_1, respectively). We have

$$\frac{x_1}{x_2} = \frac{\alpha_{21} k_D^2 + \alpha_{11} k_D + \alpha_{01}}{\alpha_{22} k_D^2 + \alpha_{12} k_D + \alpha_{02}} \tag{19}$$

In the region of the first crossover we can neglect the terms of order k_D^2 in Eq. (19), so that

$$\frac{x_1}{x_2} \approx \frac{\alpha_{11} k_D + \alpha_{01}}{\alpha_{12} k_D + \alpha_{02}} \tag{20}$$

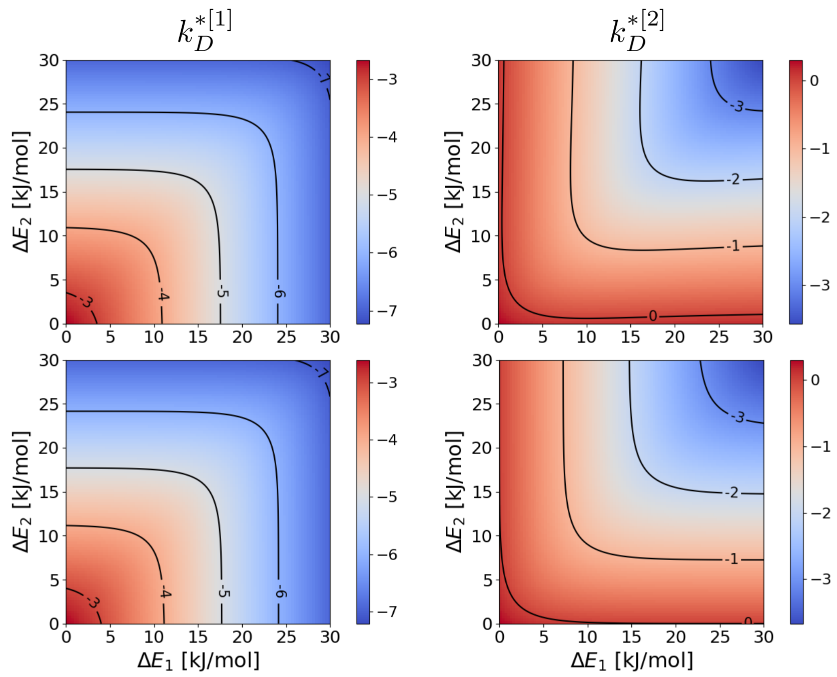

Supplementary Figure 6. Density plot of $k_D^{*[2]}$ and $k_D^{*[1]}$ in the plane $\Delta E_1, \Delta E_2$ in logarithmic (base 10) scale. The top panels correspond to the exact expressions given by Eq.(14). The bottom panel are density plots of the explicit expressions, Eq. (22) ($k_D^{*[1]}$) and Eq. (31) ($k_D^{*[2]}$). Parameters are $\eta_1 = 29.34$, $\eta_2 = 1.945$, $E_0 = 19$ kJ/mol, $E_1 = 13.6$ kJ/mol, $E_2 = 3.1$ kJ/mol, corresponding to the average values for the two furanose and pyranose enantiomers measured from equilibrium NMR experiments (see main body).

from which we can estimate $k_D^{*[1]}$ as

$$k_D^{*[1]} = \frac{\alpha_{02}}{\alpha_{12}} \tag{21}$$

After some algebraic manipulation, we have

$$k_D^{*[1]} = \frac{\mu}{P_{E_1}^{\text{eq}}(T_1)[Q(T_1) + Q(T_2)]} \tag{22}$$

where

$$Q(T) = \frac{P_{E_2}^{\text{eq}}(T)}{P_{E_0}^{\text{eq}}(T)} \left(e^{\Delta E_1/k_B T} + e^{\Delta E_2/k_B T}\right) \tag{23}$$

D. The second crossover

In this case, it is expedient to focus on the low-energy species instead (nodes 5 and 4 in the graph, corresponding to T_2 and T_1, respectively). Thus, we have

$$\frac{x_5}{x_4} = \frac{\alpha_{25}k_D^2 + \alpha_{15}k_D + \alpha_{05}}{\alpha_{24}k_D^2 + \alpha_{14}k_D + \alpha_{04}} \tag{24}$$

In the region of the second crossover we can neglect the zero-order terms in Eq. (24), so that

$$\frac{x_1}{x_2} \approx \frac{\alpha_{25}k_D + \alpha_{15}}{\alpha_{24}k_D + \alpha_{14}} \tag{25}$$

from which we can estimate $k_D^{*[2]}$ as

$$k_D^{*[2]} = \frac{\alpha_{14}}{\alpha_{24}} \tag{26}$$

The full expression in terms of the transition rates reads (see again the partial diagrams in Fig. 2)

$$k_D^{*[2]} = \frac{k_{23}k_{34}(k_{01}+k_{05}) + k_{10}k_{05}(k_{34}+k_{32}) + k_{10}k_{23}(k_{05}+k_{34}) + k_{50}k_{34}(k_{10}+k_{23})}{k_{23}k_{05}+k_{10}k_{34}} \tag{27}$$

A useful analytical expression can be derived by considering the limit $\eta_1 \to \infty$, which is seen to be a good approximation already for $\eta_1 > 0.1$. Recalling expressions (2), after some algebra, we get

$$k_D^{*[2]} = \mu \frac{G(T_1,T_2)F(T_1,T_2) + G(T_2,T_1)F(T_2,T_1)}{G(T_1,T_2)+G(T_2,T_1)} \tag{28}$$

where

$$G(T_1,T_2) = \frac{P^{\rm eq}_{E_0}(T_1)}{P^{\rm eq}_{E_1}(T_1)} e^{-\Delta E_1/T_1 - \Delta E_2/T_2} \tag{29}$$

$$F(T_1,T_2) = e^{-\Delta E_2/T_1}\left(1 + e^{(\Delta E_2 - \Delta E_1)/T_2}\right) \tag{30}$$

It turns out that formula (28) has an extremely simple and transparent approximation, namely

$$\begin{aligned} k_D^{*[2]} &\simeq \frac{\mu}{2}\left(e^{-\Delta E_1/k_BT_1} + e^{-\Delta E_2/k_BT_2} + e^{-\Delta E_2/k_BT_1} + e^{-\Delta E_1/k_BT_2}\right) \\ &= \mu\left(e^{-\Delta E_1/k_BT_M} + e^{-\Delta E_2/k_BT_M}\right) + \mathcal{O}(\Delta T^2) \end{aligned} \tag{31}$$

where we have indicated with $T_M = (T_1+T_2)/2$ the average temperature of the system and with $\Delta T = T_2 - T_1$ the temperature gradient. In figure 6 we compare the explicit analytical expressions, Eq. (22) and Eq. (31), with the exact values of the crossover rates, computed through Eqs. (14). It is seen that the two analytical estimates are in excellent agreement with the results of the full calculations, including the extremely simple formula (31).

SUPPLEMENTARY NOTE 6. CYCLE FLUXES

With reference to Fig. 2, there are three cycle fluxes in our system. According to the general diagrammatic prescription described in Ref. [19], the ratio between the one-way cycle fluxes (according to the chosen convention for positive fluxes) can be computed rather straightforwardly. More precisely, one has

$$\frac{J_{a+}}{J_{a-}} = \frac{k_{32}k_{10}}{k_{23}k_{01}} = e^{(E_0-E_1)/k_BT_m} \tag{32}$$

$$\frac{J_{b+}}{J_{b-}} = \frac{k_{43}k_{05}}{k_{34}k_{50}} = e^{-(E_0-E_2)/k_BT_m} \tag{33}$$

$$\frac{J_{c+}}{J_{c-}} = \frac{k_{43}k_{32}k_{10}k_{05}}{k_{34}k_{23}k_{01}k_{50}} = e^{-(E_1-E_2)/k_BT_m} \tag{34}$$

where

$$\frac{1}{T_m} = \frac{1}{T_1} - \frac{1}{T_2} \tag{35}$$

$$J_a = J_{a+} - J_{a-} = J_{a-}\left[e^{(E_0-E_1)/k_BT_m} - 1\right] \geq 0 \tag{36}$$

$$J_b = J_{b+} - J_{b-} = J_{b-}\left[e^{-(E_0-E_2)/k_BT_m} - 1\right] \leq 0 \tag{37}$$

$$J_c = J_{c+} - J_{c-} = J_{c-}\left[e^{-(E_1-E_2)/k_BT_m} - 1\right] \leq 0 \tag{38}$$

The above inequalities follow directly from the strict positivity of one-way cycle fluxes, which are rational functions of products of rates [19].

Much insight can be gained by inspecting the *transition* fluxes, $J_{ij} = -J_{ji}$, between any two neighboring states in the diagram. These are simply given by the sum (including the appropriate sign) of cycle fluxes for those cycles that comprise the link ij (from i to j). For example, the flux between the nodes representing the linear species from hot to cold is $J_{03} = J_a - J_b$. It should be noted that, at least in principle, transition fluxes are observable, while cycle fluxes are not.

In general, if a network comprises N nodes and M links, there are $N_J = M - N + 1$ independent transition fluxes. In our case, $M = 7$, $N = 6$, hence $N_J = 2$. These can be conveniently identified as

$$J_1 = J_a + J_c \tag{39}$$

$$J_2 = J_b + J_c \tag{40}$$

so that all transition fluxes are determined as $J_{32} = J_{21} = J_{10} = J_1$, $J_{05} = J_{54} = J_{43} = J_2$, $J_{03} = J_1 - J_2$. Note that the second law of thermodynamics is obviously not violated in the

non-equilibrium steady state, as the net energy flux still proceeds from the hot to the cold box. Recalling the definition of transition fluxes and Eqs. (39), (40), the total heat flux from the hot source to the cold one reads

$$\begin{aligned}\dot{Q} &= E_0 J_{03} + E_1 J_{12} + E_2 J_{54} \\ &= (E_0 - E_1) J_1 - (E_0 - E_2) J_2 \\ &= (E_2 - E_1) J_2 + (E_0 - E_1)(J_1 - J_2) \geq 0 \end{aligned} \tag{41}$$

where the last passage follows from the definitions of cycle fluxes (36), (37) and (38), which imply $J_2 \leq 0$, $J_1 - J_2 \geq 0$. The currents circulating in the system in the limit $k_D \to \infty$ are illustrated in Fig. 8.

SUPPLEMENTARY NOTE 7. RATE OF ENTROPY PRODUCTION AND ENERGY DISSIPATION

It is interesting to compute the rate of entropy production and to relate it to the non-equilibrium stabilisation sustained by steady currents in the system, as illustrated in the main text. The rate of entropy production can be computed as [20]

$$\dot{S} = \frac{1}{2} \sum_{i,j=0}^{5} J_{ji} \log\left(\frac{k_{ji} x_j}{k_{ij} x_i}\right) \tag{42}$$

where $J_{ji} = k_{ji} x_j - k_{ij} x_i$ is the transition flux from node j to node i. Recalling Eqs. (32), (33) and (34) and taking into account the definitions of the operational fluxes J_1 and J_2, Eqs. (39) and (40), it is not difficult to see that Eq. (42) simplifies to

$$\dot{S} = \frac{1}{k_B T_m} \left[J_1 (E_0 - E_1) - J_2 (E_0 - E_2) \right] \tag{43}$$

where T_m is the reduced temperature defined by Eq. (35). Direct inspection of Eq. (41), shows that the rate of entropy production is proportional, as it should, to the net heat flux flowing from the hot reservoir to the cold one, namely

$$k_B \dot{S} = \left(\frac{1}{T_1} - \frac{1}{T_2}\right) \dot{Q} \tag{44}$$

From the analysis of fluxes reported in the previous section, it is not difficult to realise that (see again Fig. 2) and Fig. 5 in the main text) in general the following equalities hold (at

any time)

$$J_1 = k_D(x_2 - x_1)$$
$$J_2 = k_D(x_5 - x_4) \tag{45}$$

Using Eqs. (18) and observing that $\alpha_{22} = \alpha_{21}$ and $\alpha_{25} = \alpha_{24}$ (see again the topology of the spanning trees shown in Fig. 2), we get

$$J_1 = k_D \frac{(x_2^{\rm int} - x_1^{\rm int})k_D^{*[2]}k_D + (x_2^{\rm eq} - x_1^{\rm eq})k_D^{*[1]}k_D^{*[2]}}{k_D^2 + k_D^{*[2]}k_D + k_D^{*[1]}k_D^{*[2]}}$$
$$J_2 = k_D \frac{(x_5^{\rm int} - x_4^{\rm int})k_D^{*[2]}k_D + (x_5^{\rm eq} - x_4^{\rm eq})k_D^{*[1]}k_D^{*[2]}}{k_D^2 + k_D^{*[2]}k_D + k_D^{*[1]}k_D^{*[2]}} \tag{46}$$

Plugging Eqs. (46) in the definition of the rate of entropy production, Eq. (43), the latter can be cast in the particularly transparent form

$$\dot{S} = \frac{\dot{S}_\infty k_D^2 + \dot{S}_{\rm int} k_D k_D^{*[2]}}{k_D^2 + k_D^{*[2]}k_D + k_D^{*[1]}k_D^{*[2]}} \simeq \begin{cases} \dot{S}_{\rm int}\dfrac{k_D}{k_D + k_D^{*[1]}} & \text{for} \quad k_D \ll k_D^{*[2]} \\ \dfrac{\dot{S}_\infty k_D + \dot{S}_{\rm int} k_D^{*[2]}}{k_D + k_D^{*[2]}} & \text{for} \quad k_D \gg k_D^{*[1]} \end{cases} \tag{47}$$

In particular, we get the following simple asymptotic forms,

$$\dot{S} \simeq \begin{cases} \dot{S}_{\rm int}\left(\dfrac{k_D}{k_D^{*[1]}}\right) & \text{for} \quad k_D \to 0 \\ \dot{S}_\infty \left[1 - \dfrac{k_D^{*[2]}}{k_D}\left(1 - \dfrac{\dot{S}_{\rm int}}{\dot{S}_\infty}\right)\right] & \text{for} \quad k_D \to \infty \end{cases} \tag{48}$$

E. The limit of vanishing transport rate

In the limit $k_D \to 0$, we see from Eq. (45) that the two operational fluxes read

$$J_1 \simeq \frac{1}{2} k_D \left(P_{E_1}^{\rm eq}(T_1) - P_{E_1}^{\rm eq}(T_2)\right)$$
$$J_2 \simeq \frac{1}{2} k_D \left(P_{E_2}^{\rm eq}(T_2) - P_{E_2}^{\rm eq}(T_1)\right) \tag{49}$$

where we have used Eqs. (7). Hence, we have from expression (43)

$$\dot{S} = \frac{1}{k_B T_m}\left[(E_0 - E_1)\left(P_{E_1}^{\rm eq}(T_1) - P_{E_1}^{\rm eq}(T_2)\right) - (E_0 - E_2)\left(P_{E_2}^{\rm eq}(T_2) - P_{E_2}^{\rm eq}(T_1)\right)\right] k_D \tag{50}$$

We see that the entropy production rate increases linearly with the transport rate k_D at small values of the latter. The expression (50) can be simplified further and made more transparent

by expanding it in powers of $\Delta T/T_M$, where $T_M = (T_1 + T_2)/2$ (≈ 0.85 with the choice of temperatures considered in this work, and recalling that $P^{\rm eq}_{E_0}(T_i) \ll P^{\rm eq}_{E_1}(T_i), P^{\rm eq}_{E_2}(T_i)$, $i = 1, 2$. After a straightforward calculation, we get

$$\dot{S} = \frac{1}{2} P^{\rm eq}_{E_1}(T_M) P^{\rm eq}_{E_2}(T_M) \left(\frac{E_2 - E_1}{k_B T_M} \right)^2 \left(\frac{\Delta T}{T_M} \right)^2 k_D + \mathcal{O}(k_D^2, \Delta T^3) \tag{51}$$

Fig. 9 shows the trend of the entropy production rate $\dot{S}$ as a function of the transport rate k_D for different choices of the barriers ΔE_1 and ΔE_2. It can be appreciated that the system dissipates more the faster the transport, until a maximum dissipation rate is reached that depends only on the choice of the barriers and the imposed temperature gradient.

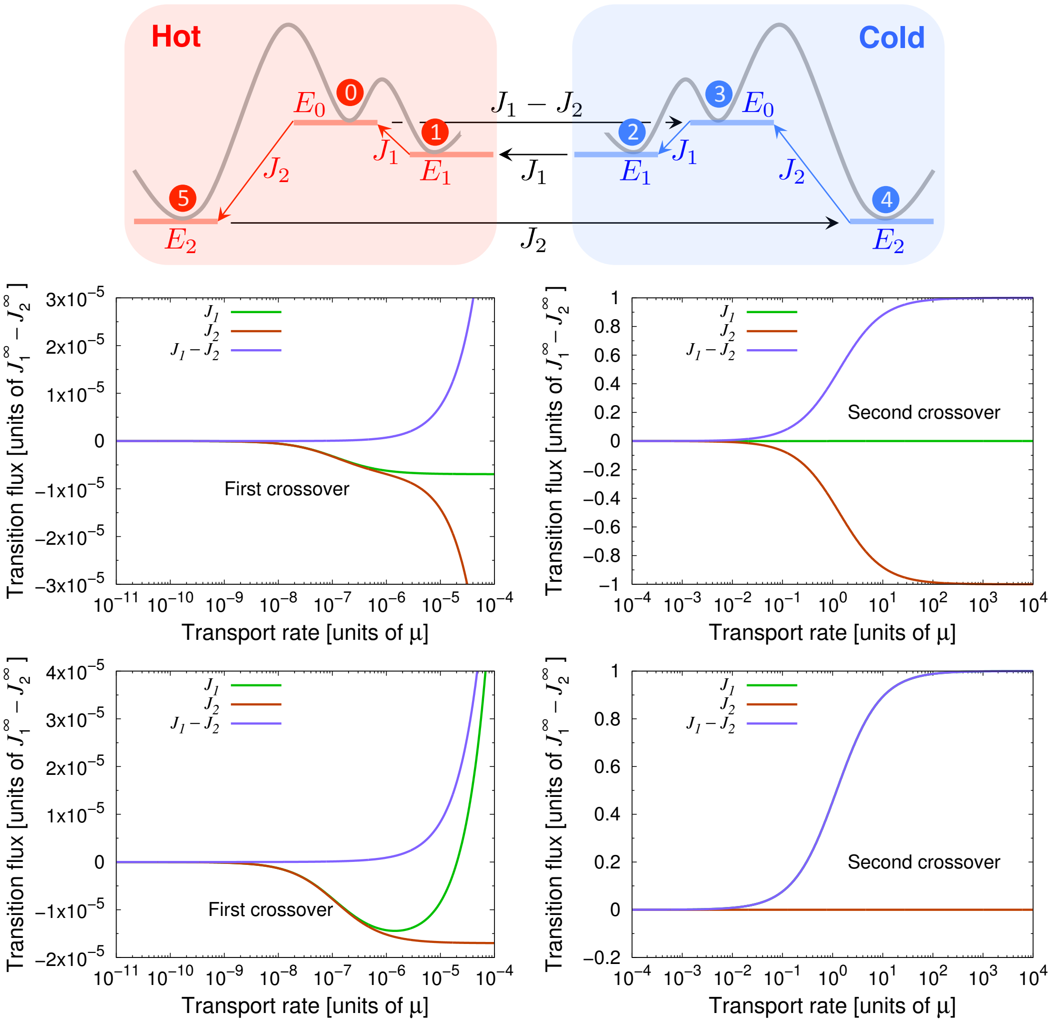

Supplementary Figure 7. The steady-state populations are sustained by steady currents, which circulate in different subgraphs in the first and second crossover regimes. Fluxes as a function of the mobility rate. Top graphs: ultra-destabilisation scenario, $\Delta E_1 = 30$ kJ/mol, $\Delta E_2 = 0$. A steady clockwise flux (b cycle, $J_1 - J_2 = -J_2 > 0$) sustains as $k_D \to \infty$ a ultra-high population of the low-energy state E_2 and an ultra-low population of the high-energy state E_1. Bottom graphs, ultra-stabilisation scenario, $\Delta E_1 = 0$, $\Delta E_2 = 30$ kJ/mol. A steady counterclockwise flux (a cycle, $J_1 - J_2 = J_2 > 0$) sustains as $k_D \to \infty$ an ultra-low population of E_2 and an ultra-high population of the E_1. Clockwise from top to bottom: $\Delta E_1 = 0$ and varying $\Delta E_2 \in [0, 30]$ kJ/mol. $\Delta E_1 = 10$ kJ/mol and varying $\Delta E_2 \in [0, 30]$ kJ/mol (the blue dashed line corresponds to $\Delta E_2 = \Delta E_1 = 10$ kJ/mol). $\Delta E_2 = 0$ kJ/mol and varying $\Delta E_1 \in [0, 30]$ kJ/mol. Varying $\Delta E_1 = \Delta E_2 \in [0, 30]$ kJ/mol. Parameters are $\eta_1 = 29.34$, $\eta_2 = 1.945$, $E_0 = 19$ kJ/mol, $E_1 = 13.6$ kJ/mol, $E_2 = 3.1$ kJ/mol, corresponding to the average values for the two furanose and pyranose enantiomers measured from equilibrium NMR experiments.

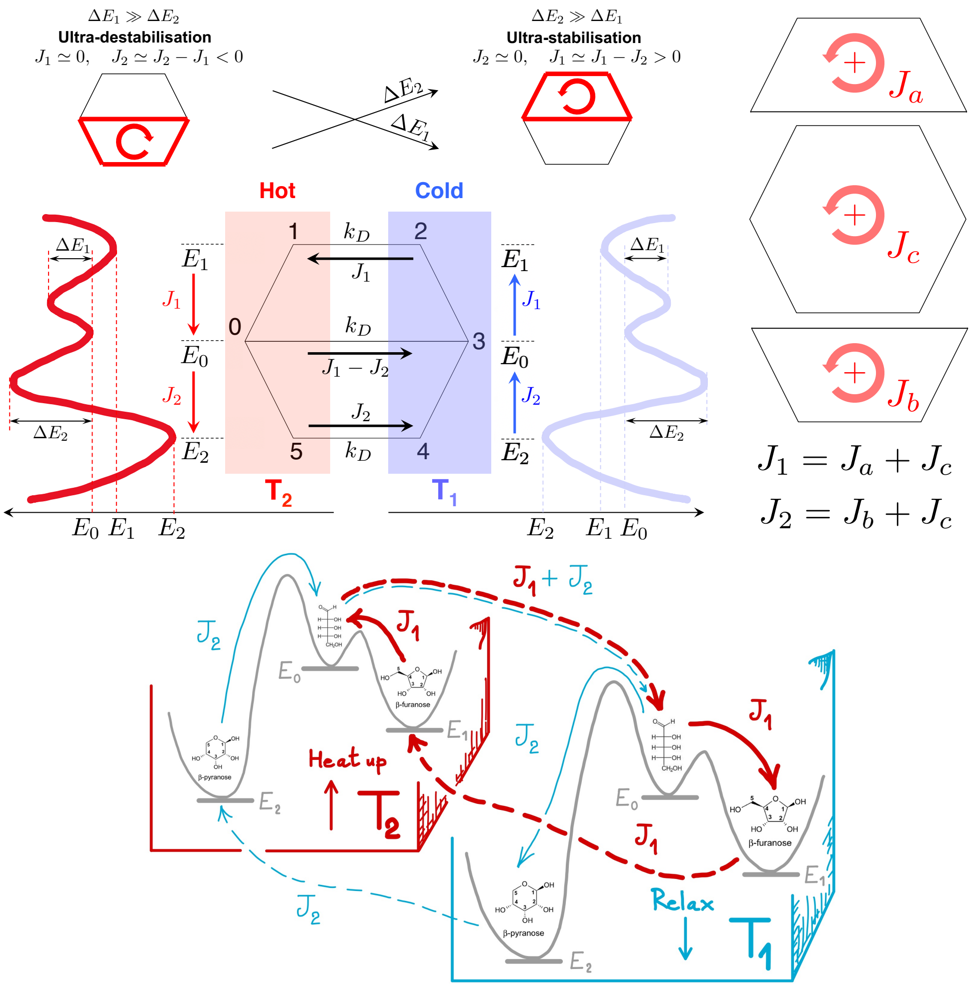

Supplementary Figure 8. **Coarse-grained ribose isomerisation network in a steady temperature gradient**. Top: as one of the linear-to-cycle transition becomes faster than the other, the corresponding barrier becoming smaller, its population is boosted as a result of sustained non-equilibrium currents, as shown within the corresponding sub-graphs. Bottom left: scheme of the network with the corresponding energy landscapes and transition fluxes. The latter can be conveniently expressed in terms of two independent linear combinations of the three cycle fluxes J_a, J_b and J_c (right). The direction of the transition fluxes shown reflects the choice made for their definition, that is, an arrow from i to j stands for the $i \to j$ flux (which can be either positive or negative). Bottom. Illustration of the steady system of currents that circulate in the system in the case of ultra-stabilisation, $\Delta E_2 \gg \Delta E_1$. Dashed arrows denote transport, solid lines stand for chemical transformations. The current J_2 is much smaller than J_1 in the fast transport limit, $k_D \gg k_D^{*[2]}$. For example, for $\Delta E_2 = 30$ kJ/mol and $\Delta E_1 = 0$, $J_2/J_1 \simeq 6.5 \times 10^{-5}$, while for $\Delta E_2 =$ 20 kJ/mol and $\Delta E_1 = 5$ kJ/mol, $J_2/J_1 \simeq 1.2 \times 10^{-2}$

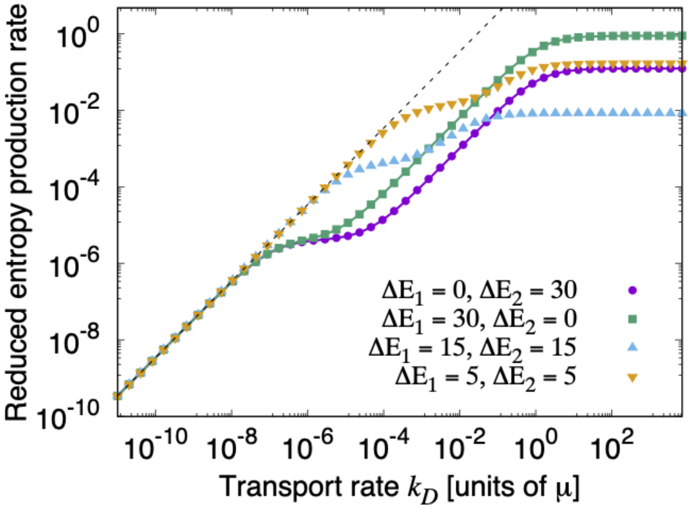

Supplementary Figure 9. Plot of the reduced entropy production rate for different choices of the two barriers ΔE_1 and ΔE_2. The solid lines are fits with the expression (47). The vanishing transport prediction (50) is shown explicitly as a dashed line. All other parameters are fixed as described in the main text at the values determined experimentally for the ribose isomerisation network.

SUPPLEMENTARY REFERENCES

[1] A. V. Dass, *Stochastic Prebiotic Chemistry*, Ph.D. thesis, Health, Biological Sciences and Chemistry of Life (SSBCV) (2018).

[2] M. A. Zharkov, *History of Paleozoic salt accumulation* (Springer-Verlag, Berlin, 1981).

[3] L. P. Knauth, Nature **395**, 554 (1998).

[4] C. E. De Ronde, D. M. Channer, K. Faure, C. J. Bray, and E. T. Spooner, Geochimica et Cosmochimica Acta **61**, 4025 (1997).

[5] H. Rollinson, *Early Earth Systems: A Geochemical Approach* (Wiley-Blackwel, 2007).

[6] L. P. Knauth, Palaeogeography, Palaeoclimatology, Palaeoecology **219**, 53 (2005).

[7] J. D. Rimstidt, Geochimica et Cosmochimica Acta **61**, 2553 (1997).

[8] K. S. Habicht, M. Gade, B. Thamdrup, P. Berg, and D. E. Canfield, Science **298**, 2372 (2002).

[9] D. Y. Sumner, American Journal of Science **297**, 455 (1997).

[10] S. A. Crowe, C. A. Jones, S. Katsev, C. Magen, A. H. O'Neill, A. Sturm, D. E. Canfield, G. D. Haffner, A. Mucci, B. Sundby, and D. A. Fowle, Proceedings of the National Academy of Sciences of the United States of America **105**, 15938 (2008).

[11] H. D. Holland, B. Lazar, and M. McCaffrey, Nature **320**, 27 (1986).

[12] V. Busigny, N. J. Planavsky, D. Jézéquel, S. Crowe, P. Louvat, J. Moureau, E. Viollier, and T. W. Lyons, Geochimica et Cosmochimica Acta **133**, 443 (2014).

[13] E. Douville, J. L. Charlou, E. H. Oelkers, P. Bienvenu, C. F. Jove Colon, J. P. Donval, Y. Fouquet, D. Prieur, and P. Appriou, Chemical Geology **184**, 37 (2002).

[14] J. A. Hawkes, D. P. Connelly, M. Gledhill, and E. P. Achterberg, Earth and Planetary Science Letters **375**, 280 (2013).

[15] R. M. Gallant and K. L. Von Damm, Geochemistry, Geophysics, Geosystems **7** (2006).

[16] D. E. Canfield, Annu. Rev. Earth Planet. Sci **33**, 1 (2005).

[17] A. Kappler, C. Pasquero, K. O. Konhauser, and D. K. Newman, Geology , 865 (2005).

[18] A. Ricardo, M. A. Carrigan, A. N. Olcott, and S. A. Benner, Science **303**, 196 (2004).

[19] T. L. Hill, *Free Energy Transduction and Biochemical Cycle Kinetics* (Springer-Verlag New York, 1989).

[20] J. Schnakenberg, Reviews of Modern Physics **48**, 571 (1976).
